# Supplementary material for: Transfer learning for non-image data in clinical research: A scoping review
Source: PLOS Digit Health. 2022 Feb 17;1(2):e0000014. doi: 10.1371/journal.pdig.0000014 (PMC9931256; doi:10.1371/journal.pdig.0000014)
Supplement: S1 Glossary — (PDF) [file pdig.0000014.s005.pdf]

Artificial intelligence (AI)
A non-human program or model that can solve sophisticated tasks.

Machine learning (ML)
The training of programs developed by allowing a computer to learn from its experience, rather than through manually coding individual steps.

Neural network
A neural network is a particular kind of ML model. A model that, taking inspiration from the brain, is composed of layers of neurons. An input layer (first layer) receives the input data. This is followed by hidden layer(s). Hidden neurons (yellow circles) typically take multiple input values and generate one output value, which is calculated by applying a nonlinear transformation (activation function) to a weighted sum of input values. An output layer (final layer) returns the results. Model fitting (training) aims to optimize the weights to get the best model according to a pre-defined performance metric (loss).

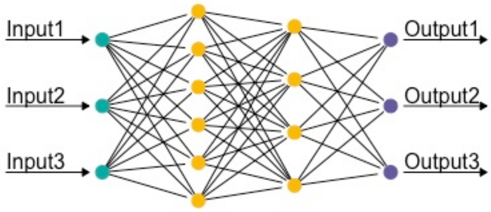

Deep learning
A type of machine learning that uses neural networks with multiple hidden layers.

Transfer learning
The use of a pre-trained model for a task different to what it was originally trained for. For example, to take a model that can recognize everyday objects and further train it on fundus photographs to grade diabetic retinopathy, instead of training a model from scratch on fundus photographs only.

Fine-tuning
A type of transfer learning that updates the parameters of a pre-trained model by training for a different task than it was originally trained for.

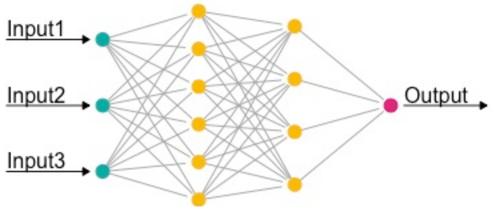

Feature-representation transfer
A transfer learning technique that passes input data through a pre-trained model and extracts feature-representations (values from hidden layers), which then become inputs for another model for a different task than the pre-trained model was trained for.

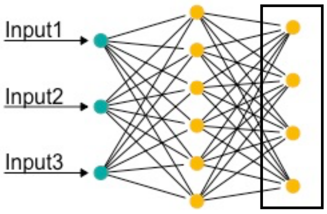

Common concepts with different names in clinical research and computer science:

- fit a model = train a model, network
- covariate, variable = feature
- outcome = target
- coefficient = weight, parameter

References
1. Howard J, Gugger S. Deep Learning for Coders with fastai and PyTorch. 1 ed: O'Reilly Media, Inc.; 2020.
2. Google. Machine Learning Glossary 2021 [Available from: https://developers.google.com/machine-learning/glossary]
