# Supplementary material for: Transfer learning for non-image data in clinical research: A scoping review
Source: PLOS Digit Health. 2022 Feb 17;1(2):e0000014. doi: 10.1371/journal.pdig.0000014 (PMC9931256; doi:10.1371/journal.pdig.0000014)
Supplement: Attachment — Submitted filename: TLScR_Response_R1.docx [file pdig.0000014.s006.docx]

Reviewer #1: This manuscript provides a survey of the current utilization of transfer learning in clinical studies which focus on non-image applications. The authors focus on clinical journals rather than technical or interdisciplinary venues (e.g., "Machine Learning for Healthcare" ([https://www.mlforhc.org/](https://www.mlforhc.org/)) is not included). In general, I think such a survey is a useful contribution and highlights several gaps/lags between the general machine learning and clinical research communities.

My primary concern with the manuscript in its current form is a potentially too restrictive set of inclusion criteria, which does not consider several (arguably valid) common use cases of transfer learning, particularly in the NLP/text modality.

We thank the reviewer for the kind words and the constructive feedback. We have provided our responses to each comment/question below.

Comments/Questions
------------------------------------
"Finally, we also excluded studies which combined source data with the target data as an integral part of their analysis as opposed to reusing only a source model."

This strikes me as unnecessarily strict a requirement. Augmenting a source model in some fashion using source+target data is quite reasonable and is a beneficial application of transfer learning. Can the authors elaborate on their rational here?

We agree with the reviewer in that combining source and target data can also be a relevant form of transfer learning. However, clinical researchers often struggle with data access due to privacy issues, which can be a barrier for collaborative efforts. We see a huge potential in the two included forms of transfer learning (i.e. fine-tuning and feature representation transfer) to overcome this barrier, and therefore focused on these in the scoping review. We have now added a section to the limitations, including an example (Desautels 2017) and explaining why we decided not to include this form of transfer learning.

[Inserted at the beginning of the 'Limitations' section]

*'In our study protocol, we choose to exclude studies combining source and target data (n=30) from our review, even though we acknowledge the value of this approach in certain scenarios [e.g. Desautels 2017]. We felt, however, that current data sharing restrictions would present barriers to the practical use and clinical impact of this type of transfer learning. Fine-tuning and feature-representation transfer, on the other hand, can aid to overcome such barriers by reusing models instead of the actual data, which is why these two forms of transfer learning were in the focus of our review.'*

------------------------------------
The inclusion/exclusion criteria aren't entirely clear to me and for findings for studies using transfer learning for text ("text (n=7; 8%)") seem far too low.

For example, these papers are all findable by PubMed and seem, at first cut, to use transfer learning and pass the 5 inclusion criteria outlined on page 6 of the manuscript.

- "Classifying Alzheimer's Disease Using Audio and Text-Based Representations of Speech" (Jan 2021) [https://pubmed.ncbi.nlm.nih.gov/33519651/](https://pubmed.ncbi.nlm.nih.gov/33519651/)

- "Inferring ADR causality by predicting the Naranjo Score from Clinical Notes" (Jan 2021) [https://www.ncbi.nlm.nih.gov/pmc/articles/PMC8075501/](https://www.ncbi.nlm.nih.gov/pmc/articles/PMC8075501/)

- "Exploring Fever of Unknown Origin Intelligent Diagnosis Based on Clinical Data: Model Development and Validation" (Nov 2020) [https://pubmed.ncbi.nlm.nih.gov/33172835/](https://pubmed.ncbi.nlm.nih.gov/33172835/)

et alia.

These studies seems to reflect the criteria "be a clinical study or focus on clinically relevant outcomes or measurement". I don't quite buy the claim (page 22) that tasks like NER should be excluded since they "only indirectly relevant from a clinical perspective e.g. by speeding up knowledge synthesis." In many clinical analyses, patient outcomes are directly determined by looking for textual evidence in clinical notes, so any text mining technique that leverages some flavor of BERT to identify patient outcomes should arguably be included. Not including this common use case undercuts the manuscript's findings about the limited uptake of transfer learning for text/NLP.

Similar to the reviewer, we were also surprised by the low number of articles about NLP used for a clinical purpose. We think that this is due to a methodological challenge, rather than a limited uptake of transfer learning. We conducted a rigorous systematic search with the assistance of an experienced librarian and published our search strategy:

https://figshare.com/articles/online_resource/Transfer_learning_for_non-image_data_in_clinical_research_a_scoping_review_protocol/14638914/1

As seen in the search criteria, we included not only the term 'transfer learning', but also less often used terms, like 'knowledge transfer', 'learning to learn', etc. The three above-mentioned articles were not identified by the search (and thus were not included in the screening process), because they neither included 'transfer learning' explicitly in the article nor similar terms in the abstract.

In hindsight, one could argue that 'BERT' and other commonly used pre-trained models should have been part of our search strategy. Unfortunately, we did not have a complete list of relevant pre-trained NLP models and were reluctant to search for the name of specific models as this could skew our results (e.g. drawing a conclusion that almost all NLP studies used BERT), and instead chose a pragmatic solution. We hope our work will motivate future studies to synthetize knowledge about the application of such specific language models in clinical research, as their use is increasing rapidly. Naturally, we should have kept this limitation in mind when discussing our findings on text analyses and this section has now been revised (see below).

Regarding NER, we find topic interesting and acknowledge that is debatable if it is clinical or not, as mentioned in limitation. However, we maintain that we do not consider NER in itself to be clinical research unless a study used it for something clinically relevant (prediction, prognosis, identifying patients who should have been referred to a specific treatment, etc.). This is similar to how we handled papers on the technical aspects of improving brain computer interfaces (BCI) without a clinical application. For the readers interested specifically in NER, we have included a reference to a recent review on the topic (https://www.mdpi.com/2076-3417/11/18/8319/htm), similar to what we did for BCI studies.

[Changes to the 'Text' discussion ]:

*'~~Our main finding regarding text and natural language processing (NLP) was how underutilized transfer learning was in clinical research.~~ '*

*'It is somewhat surprising that we found so few studies, given the massive interest in NLP and the field’s rapid development in recent years [119, 120]. The main reason behind this is methodological. Our systematic search could only identify studies explicitly mentioning transfer learning or a similar term (e.g. ‘knowledge transfer’), however this does not seem to be a common practice in medical text analysis [three above-mentioned refs]. Future studies are warranted about the impact of specific models (e.g. BERT[Devlin et al ref#94 in the preprint]) on clinical research. ~~The relatively slow adoption of transfer learning in clinical research is presumably due to the many~~ There are also technical challenges in medical text analysis (e.g. ambiguous abbreviations, specialty-specific jargon, etc.) [121]. '*

[inserted in the 'Limitations' section, changes marked with **bold**]

*'Similarly, we excluded NLP studies on biomedical named entity recognition, because even though the datasets might have been medically relevant documents, the task of identifying specific terms are* ***often*** *indirectly relevant from a clinical perspective e.g. by speeding up knowledge synthesis.* ***A recent review provides an overview of the approach and its clinical applications [Bose P et al. 2021].****'*

------------------------------------

The "discussion" section is quite long and I feel some content could be moved out of this section and into "results"

We thank the reviewer for the feedback and acknowledge that the review is long. However, we think it is important to mention many different examples for the use of transfer learning to inspire clinical researchers with an interest in transfer learning. The discussion is divided into sections by the different data types, so that the readers can easier navigate to the parts that interest them. After reading the reviewer's comment, we shortened or removed some parts, especially from the 'Text' section of the discussion. If the reviewers and editor still find the discussion too long, we will naturally do our best to further shorten the discussion in a new revised version and welcome any suggestions on how best to achieve this.

[changes to ‘Results’ marked with bold]

‘Studies with pure technical affiliations were more common (29 out of 83; 35%) than studies with pure clinical affiliations (4 out of 83; 5%)**, except for studies analyzing text, where all articles were written by clinicians with or without technical co-authors**.’

[changes to discussion of ‘Text’]

’~~While transfer learning in NLP has made considerable progress from a technical perspective, it seems that this trend has only just begun to appear in clinical research~~.’

‘We can indirectly observe this in our review, where all ~~seven~~ articles were written by clinicians and technical authors together or by clinicians alone, **in contrast to other data types**. ~~This is in contrast with what we observed for time series, tabular data, and audio, where articles were more often written by technical authors only~~.’

‘~~Several articles have been written on transfer learning using radiological reports or HER~~

~~…~~

, ~~which are not indexed in the databases that clinicians are familiar with~~’

Reviewer #2: This paper reports a scoping review of transfer learning for non-image data in clinical research, discusses its trends and highlights numerous potential works.

Overall the review is well performed, with a comprehensive review of the current literature. The review is also well written, balanced and clear. This paper fills an essential gap in the current literature.

We thank the reviewer for the positive comments on our manuscript.
